# RNAi-Mediated Knockdown of Calreticulin3a Impairs Pollen Tube Growth in *Petunia*

**DOI:** 10.3390/ijms23094987

**Published:** 2022-04-30

**Authors:** Piotr Wasąg, Anna Suwińska, Marta Lenartowska, Robert Lenartowski

**Affiliations:** 1 Department of Cellular and Molecular Biology, Faculty of Biological and Veterinary Sciences, Nicolaus Copernicus University in Torun, 87-100 Torun, Poland; piotr.wasag@ukw.edu.pl (P.W.); asu@umk.pl (A.S.); mlenart@umk.pl (M.L.); 2 Department of Biochemistry and Cell Biology, Faculty of Biological Sciences, Kazimierz Wielki University, 85-093 Bydgoszcz, Poland; 3 Centre for Modern Interdisciplinary Technologies, Nicolaus Copernicus University in Torun, 87-100 Torun, Poland

**Keywords:** calreticulin, molecular cloning, phylogenetic analysis, siRNA, pollen tube, actin cytoskeleton, molecular chaperoning, calcium homeostasis, in vitro studies, *Petunia hybrida*

## Abstract

Pollen tube growth depends on several complex processes, including exo/endocytosis, cell wall biogenesis, intracellular transport, and cell signaling. Our previous results provided evidence that calreticulin (CRT)—a prominent calcium (Ca^2+^)-buffering molecular chaperone in the endoplasmic reticulum (ER) lumen—is involved in pollen tube formation and function. We previously cloned and characterized the *CRT* gene belonging to the *CRT1/*2 subgroup from *Petunia hybrida* (*PhCRT1/2*), and found that post-transcriptional silencing of *PhCRT1/2* expression strongly impaired pollen tube growth in vitro. Here, we report cloning of a new *PhCRT3a* homolog; we identified the full-length cDNA sequence and described its molecular characteristics and phylogenetic relationships to other plant *CRT3* genes. Using an RNA interference (RNAi) strategy, we found that knockdown of *PhCRT3a* gene expression caused numerous defects in the morphology and ultrastructure of cultivated pollen tubes, including disorganization of the actin cytoskeleton and loss of cytoplasmic zonation. Elongation of si*PhCRT3a* pollen tubes was disrupted, and some of them ruptured. Our present data provide the first evidence that *PhCRT3a* expression is required for normal pollen tube growth. Thus, we discuss relationships between diverse CRT isoforms in several interdependent processes driving the apical growth of the pollen tube, including actomyosin-dependent cytoplasmic streaming, organelle positioning, vesicle trafficking, and cell wall biogenesis.

## 1. Introduction

**Figure 1:**
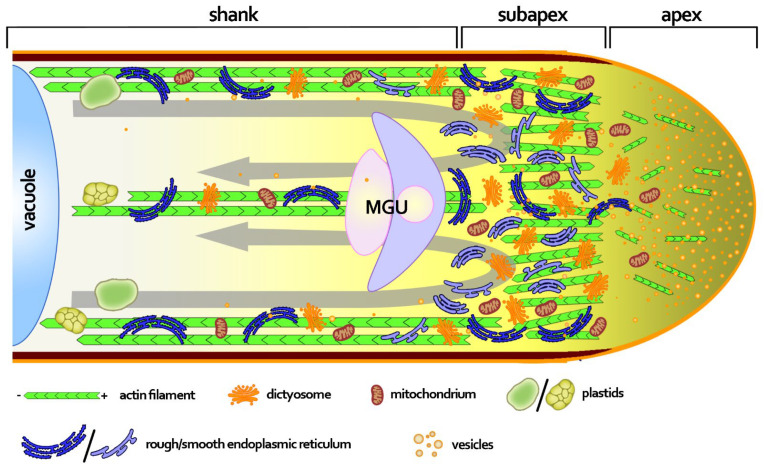
A schematic representation of the angiosperm growing pollen tube illustrating three distinct zones: apex, subapex, and shank. The apex is packed with transport vesicles and void of large organelles, leading to the formation of a “clear zone” with a network of fine and highly dynamic microfilaments. Behind the actin fringe at the subapical zone of the tube, F-actin forms strong actin bundles required for transport processes. Vesicles and organelles, such as mitochondria, endoplasmic reticulum (ER), and dictyosomes, are transported on cortical actin bundles towards the growing tip. Retrograde transport takes place on central actin cables, resulting in a “reverse fountain” pattern of cellular transport (grey arrows). Vesicles and small organelles are present throughout the pollen tube’s cytoplasm, but their highest concentration is in the subapical region. Large organelles, such as plastids and vacuoles, are localized in the shank. The biochemical composition of the pollen tube cell wall changes along the longitudinal axis; pectins predominantly form the primary cell wall at the tip, whereas further back the cell wall is composed of pectins, cellulose, and callose (brown), which is absent from the apex, leading to increased stiffness of the shank’s cell wall. Yellow intensity from the apex to the shank illustrates the tip-focused calcium (Ca^2+^) gradient in the pollen tube’s cytoplasm. MGU: male germ unit. Not to scale.

In flowering plants, the transport of non-motile sperm cells to the embryo sac is carried out by the pollen tube—a highly specialized tip-growing cell generated by the pollen grain. Indeed, maintaining the unique tubular shape of the pollen tube, which extends through the pistil-transmitting tract, is essential for successful fertilization [1]. In addition to its crucial role in sexual plant reproduction, the pollen tube is also an excellent model for studying many aspects of plant cell biology in in vitro cultures. Cultivated pollen tubes can be easily manipulated using molecular biology tools, allowing detailed studies of the key cellular processes/mechanisms involved in rapid apical growth, such as maintaining the balance between exocytosis and endocytosis, biogenesis of the cell wall, and intracellular transport driven by the cytoskeleton. The polar growth of a pollen tube is closely related to the tube’s internal ultrastructure, and an elongated tube can be divided into three main zones (Figure 1): a hemispheric apex, a relatively short subapex, and a very long shank [2].

The apical domain is usually called the “clear zone” because it almost entirely lacks larger organelles, including light-refracting amyloplasts [3]. This zone contains an inverted cone of transport vesicles that fuse with the growing tip to provide the membrane and cell wall extensions. A large amount of evidence supports the classical model of pollen tube tip growth, proposing apical secretion and lateral endocytosis. However, this model was recently challenged based on studies indicating that lateral exocytosis may be balanced by apical endocytosis [4,5]. The apical domain is also the tube region able to perceive external signals and to uptake water, ions, and nutrients. Mitochondria and dictyosomes are present throughout the pollen tube’s cytoplasm, but their highest concentration is found in the subapical region [2]. This domain is also enriched in the tubular (smooth) endoplasmic reticulum (ER), whereas the rough ER is abundant, starting behind the inverted cone of vesicles throughout the rest of the cytoplasm. Proximal to the subapical zone, the distal shank elongates gradually due to progressive exocytosis at the apical dome, and becomes a biological transport route of the male germ unit (MGU), consisting of the vegetative nucleus and two sperm cells. Numerous large organelles, such as plastids and vacuoles, are also present in the shank. The biochemical composition of the pollen tube’s cell wall changes along the longitudinal axis. At the growing tip of the angiosperm pollen tube, a primary cell wall is mostly composed of pectins, providing this zone with elastic properties that allow directional expansion [6]. In contrast, the cell wall of the tube shank consists of a primary cell wall composed of polysaccharides (pectins and cellulose) and a secondary cell wall (an inner layer) composed of callose, which is absent from the apex of angiosperm pollen tubes.

A large amount of evidence has demonstrated that the actin cytoskeleton plays a crucial role in driving pollen tube growth and morphogenesis by providing actomyosin-dependent cytoplasmic streaming and vesicular trafficking in the apical dome [7,8,9]. The actin filaments are arrayed into three distinct populations in growing pollen tubes, consistent with the zonation of the cytoplasm (Figure 1)—long and thick actin bundles in the shank, a cortical actin fringe in the subapex, and a network of fine microfilaments at the apex. Electron microscopy revealed that longitudinally aligned actin bundles show uniform polarity in the shank, but the cortical actin filaments and the inner actin filaments have their barbed ends facing toward the tip and base of the pollen tube, respectively [10]. The actin cables in the shank are important for a transport mechanism in angiosperm pollen tubes called reverse-fountain cytoplasmic streaming, whereby the cytoplasm flows toward the tip along the edge of the tube and toward the base through the center of the tube. This mechanism allows transport of organelles and vesicles to the growing tip; however, large organelles do not enter the subapical zone, whereas small organelles and vesicles enter it and become accumulated there [3]. At the base of the clear zone, the cytoplasmic stream reverses direction, and most of the vesicles are captured by the actin fringe. Visualization of this unique actin structure in growing pollen tubes (also called collar or funnel) has been optimized with the development of improved methods of fixation [11], or by using transformed pollen tubes expressing actin-binding proteins fused with fluorescent markers [12]. The actin fringe, together with highly dynamic microfilaments at the apex, controls vesicle docking and fusion events in the apical dome [8].

The pollen tube’s growth and shape are controlled by a complex network of cellular events and factors, and the most recent review by Çetinbaş-Genç et al. [13] indicates that the misfunction of a single component causes serious defects during the tube’s elongation. One of the important factors in pollen germination and pollen tube growth is calcium (Ca^2+^). The growing pollen tube possesses a tip-focused gradient of cytosolic Ca^2+^, which is closely related to several interdependent processes driving pollen tube elongation [14]. On the other hand, a very high rate of protein synthesis and protein folding in the ER is strictly required in a rapidly growing pollen tube. Thus, we hypothesized that one of the key molecules controlling Ca^2+^ homeostasis and molecular chaperoning in elongating pollen tubes is calreticulin (CRT). This Ca^2^^+^-binding/buffering molecular chaperone is translated on membrane-bound ribosomes, and accumulates in the abundant ER in the subapical zone of *Petunia* pollen tubes [15]. Two distinct groups of CRT isoforms have been distinguished in plants: CRT1/2 and CRT3; these proteins play important roles in a variety of cellular processes, including Ca^2+^ signaling and protein folding [16,17,18,19]. However, while the three different CRT family members retain basic CRT functions related to Ca^2+^ homeostasis and quality control of newly synthesized glycoproteins in the ER, they also have diverged functions in plants. CRT1/2 isoforms appear to work as primary proteins within a general ER chaperone framework, whereas plant-specific CRT3 seems to be co-expressed with pathogen/signal-transduction-related genes [16,17,18,19]. We previously cloned and characterized the *PhCRT* gene from *Petunia*, belonging to the subgroup *CRT1/2* [20], and showed its elevated expression during pollen development [21] and pollen–pistil interactions [20]. Moreover, using the RNA interference (RNAi) strategy, we found that post-transcriptional silencing of *PhCRT1/2* expression strongly impaired pollen tube growth in vitro [22]. Therefore, we proposed an important role for CRT belonging to the subgroup CRT1/2 during the key reproductive events in angiosperms, including pollen tube growth. Here, we report cloning of a new *PhCRT3a* homolog; we identify the full-length cDNA sequence, and describe its molecular characteristics and phylogenetic relationships to other plant *CRT* genes. Because *PhCRT3a* is highly expressed in cultivated pollen tubes, we test its function in the tube elongation using small interfering RNA (siRNA), and discuss probable roles for *Ph*CRT3a in pollen tubes’ apical growth.

## 2. Results

### 2.1. PhCRT3a Gene Cloning, Analysis of the Deduced Amino Acid Sequence of the PhCRT3a cDNA Clone, and Phylogenetic Analysis

**Figure 2:**
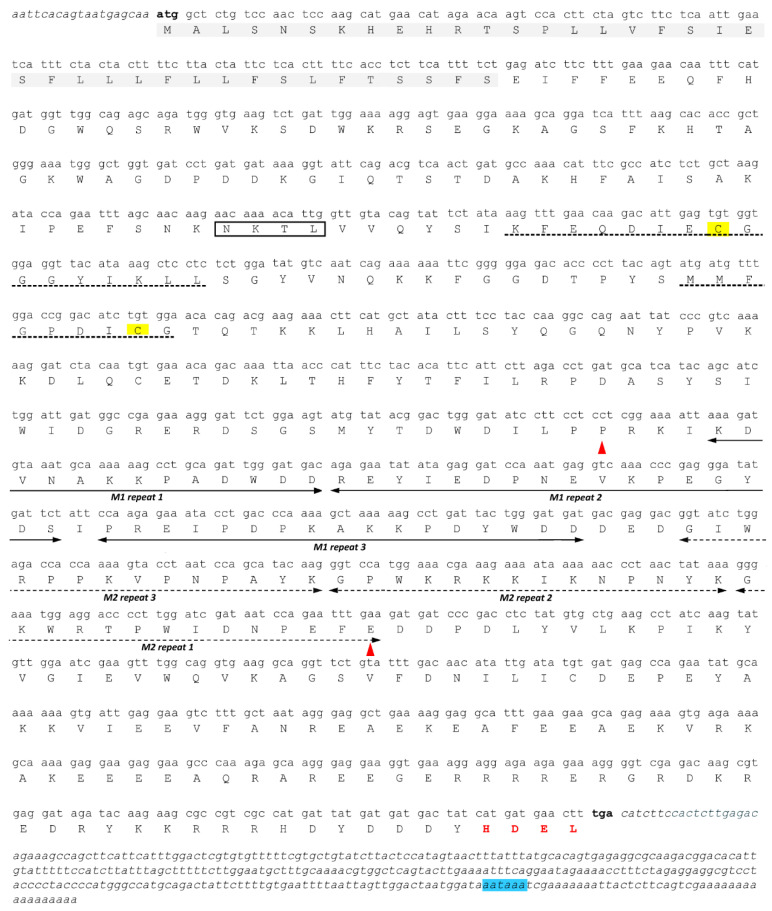
Nucleotide sequence of the full-length *Petunia hybrida* calreticulin 3a (*PhCRT3a)* cDNA and its predicted amino acid sequence. The predicted signal peptide in the N-domain is highlighted in grey, and the predicted *N*-glycosylation site is boxed. CRT family signature motifs 1 and 2 are shown by dotted lines. The triplicate repeats M1 [PXXIXDP(E/D)(A/D)XKP(E/D)DWD(D/E)] and M2 [GXWXXPXIXNPXYX] are marked with two-headed solid and two-headed dotted arrows, respectively. The cysteine residues are marked with yellow. The proline-rich P-domain is enclosed by the two red arrowheads. The ER-retention motif (HDEL) is indicated in red font in the C-terminal domain, while blue highlighting marks the polyadenylation signal. Nucleotide sequences of the 5′ and 3′ UTRs are in italics, while the start and stop codons are indicated in bold.

The 1406 bp partial *CRT3* cDNA sequence of *Petunia inflata* (Peinf101Scf01985g01011.1) published in the Sol Genomics Network was used to design gene-specific primers that were used to clone the *PhCRT3a* 3′ and 5′ ends by rapid amplification of cDNA ends. The resulting fragments were sequenced, and served as template to design a set of primers to amplify a full-length *PhCRT3a* cDNA, which was recombined into the pCR4 Blunt-TOPO vector. The *PhCRT3a* cDNA sequence is 1652 bp, and consists of a 19 bp 5′ untranslated region (UTR) upstream of the ATG initiation codon, a 1296 bp open reading frame (ORF) that terminates with a TGA stop codon, and a 320 bp 3′ UTR (Figure 2). One putative polyadenylation signal sequence (AATAAA) [23] was mapped at the 3′-end of the *PhCRT3a* sequence, 24 bp upstream of the poly(A) tail. The full-length *PhCRT3a* cDNA sequence was submitted to GenBank (accession number OM807130).

Cloned *PhCRT3a* cDNA was subjected to translation in silico, which revealed that the putative *PhCRT3* ORF encodes a slightly acidic (pI 5.98), as predicted by the Compute pI/Mw (ExPASy) tool [24] — a 431-amino-acid polypeptide with a calculated molecular mass of 50.6 kDa. Similar to other plant CRTs, the *Ph*CRT3a polypeptide has three evolutionarily conserved domains: N, P, and C (Figure 2). The globular N-domain starts with a predicted signal peptide and contains a predicted *N*-glycosylation site (NKTL), identified using NetNGlyc 1.0 software [25]. The N-domain also comprises two conserved CRT family signature motifs 1 and 2, with cysteine residues involved in disulfide bridge formation essential for proper folding of the N-terminal region [16]. The P-domain in the middle part of the *Ph*CRT3a polypeptide contains three repeats each of two proline-rich sequences — M1 [PXXIXDP(E/D)(A/D)XKP(E/D)DWD(D/E)] and M2 [GXWXXPXIXNPXYX] — which are arranged asymmetrically relative to one another. Both motifs, evolutionarily conserved among all members of the CRT family, are responsible for Ca^2^^+^-binding properties with high affinity but low capacity, as well as lectin-like chaperone activity of the plant CRTs [16,17,18]. The C-terminus of *Ph*CRT3a is enriched in negatively charged residues that are responsible for binding Ca^2^^+^ with relatively high capacity, but low affinity. We estimated that the content of acidic residues was 21.6% in the P-domain and 29.7% in the C-domain of the *Ph*CRT3a polypeptide. Finally, the C-domain ends with an HDEL sequence, which is required for the retention of plant CRTs in the ER lumen [16,17,18].

**Figure 3:**
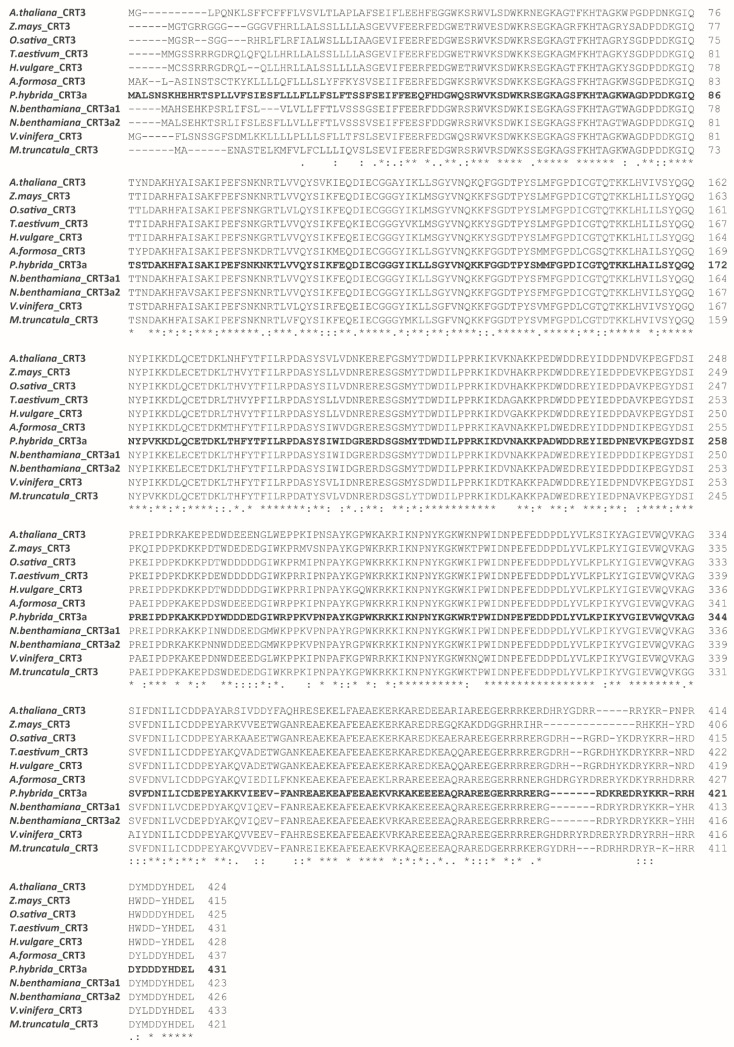
Sequence alignment of the newly identified *Ph*CRT3a with other plant CRT3 sequences: *Oryza sativa* (BAC06263.1), *Triticum aestivum* (ABR15365.1), *Zea mays* (translated from nucleotide sequence AY105822.1) [16], *Aquilegia formosa* (translated from nucleotide sequences DR940519.1 and DT750168.1), *Arabidopsis thaliana* (NP_563816.1), *Hordeum vulgare* (translated from nucleotide sequence AK248906.1), *Vitis vinifera* (translated from nucleotide sequence XM_002276397.3) [28], *Nicotiana benthamiana* [29], and *Medicago truncatula* [30]. The *Ph*CRT3a sequence was reported to the NCBI database. The numbers on the right-hand side indicate the amino acid position. Asterisks indicate a single, fully conserved residue. Colons or dots denote conservation between groups with strongly similar properties (>0.5 in the Gonnet PAM 250 matrix) or weakly similar properties (<0.5 in the Gonnet PAM 250 matrix), respectively [27].

**Figure 4:**
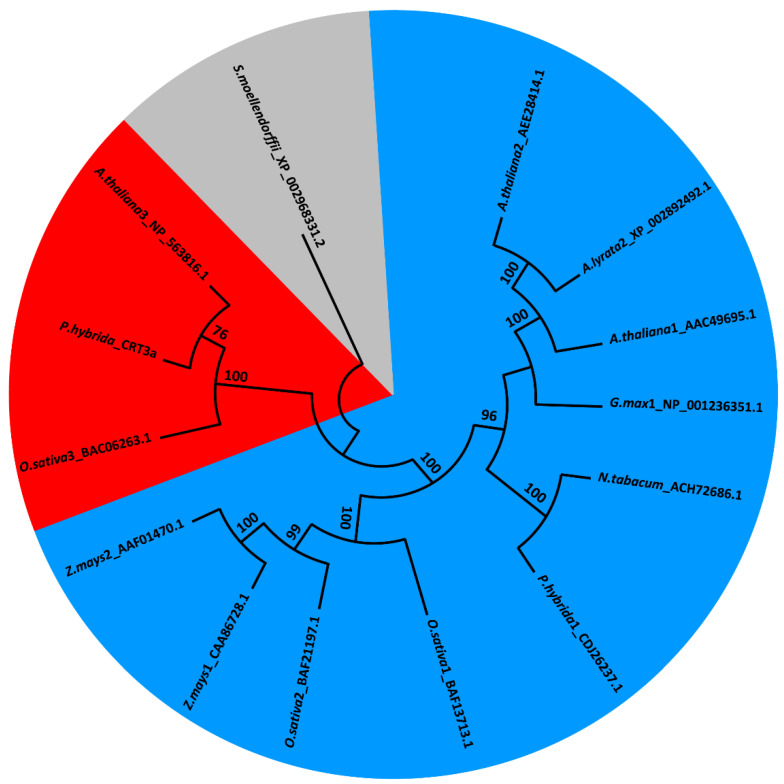
Phylogenetic analysis of selected plant CRT amino acid sequences. The analysis includes the newly identified *Ph*CRT3a and 12 isoform-verified CRTs obtained from NCBI with the following accession numbers: *Arabidopsis thaliana* (AAC49695.1; AEE28414.1; NP_563816.1), *Arabidopsis lyrata* (XP_002892492.1), *Nicotiana tabacum* (ACH72686.1), *Glycine max* (NP_001236351.1), *Oryza sativa* (BAF13713.1; BAF21197.1; BAC06263.1), *Petunia hybrida* (CDJ26237.1), and *Zea mays* (CAA86728.1; AAF01470.1). A neighbor-joining phylogenetic tree was built using Mega X. To estimate the branch support, the Poisson correction model was used. The numbers at nodes indicate percentage values of bootstraps received by 1000 replications. The tree was rooted by the *Selaginella moellendorffii* CRT sequence (XP_002968331.2). A number after a species name describes a specific CRT isoform, as do the colored areas (CRT1/2—blue, CRT3—red). The rooted sequence is marked grey.

To confirm the membership of the *Ph*CRT3a polypeptide in the plant-specific CRT3 subclass, and determine its interconnectedness with the other plant CRTs, comparative alignment analysis was performed using the EMBL-EBI search and sequence analysis tools [26,27]. For this purpose, the NCBI database was searched, and 12 isoform-verified sequences were selected. The comparative analysis using Clustal Omega revealed that the level of identity in amino acid sequences between *Ph*CRT3a and other CRT isoforms exceeded 51%. The highest discrepancies between the sequences being compared were observed at the N- and C-termini (Figure S1 in the Supplementary Materials). We also performed additional alignment of *Ph*CRT3a with other plant-specific members of the CRT3 subclass found in the NCBI database and the literature data [16,28,29,30]. The identity at the amino acid level in this case was very high — between 75% and 87% for *Zea mays* and *Nicotiana benthamiana*, respectively (Figure 3). Finally, we created a neighbor-joining tree based on the Poisson correction model (Figure 4). The cladogram analysis shows a clear distance between diverse subclasses of the CRT isoforms. All of the CRT1/2 sequences used in our analysis were clustered into one common clade, while *Ph*CRT3a was grouped with the other CRT3 isoforms, confirming its membership of this plant-specific subclass. Importantly, for each node, the bootstrap values (received by 1000 replicates) were extremely high — above 96% [31]; just one value was 76%. As an outgroup we used an evolutionarily distant sequence derived from *Selaginella moellendorffii*.

### 2.2. Knockdown of PhCRT3a Expression Impairs Pollen Tube Elongation

**Figure 5:**
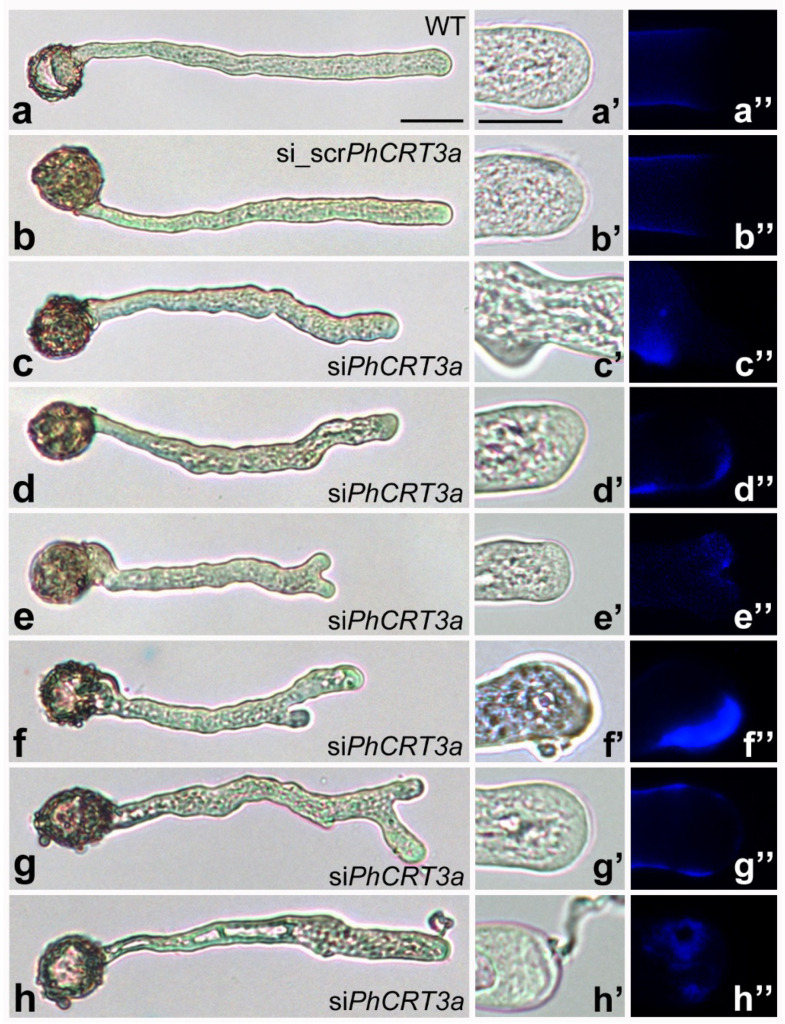
Morphology and callose deposition of/in *Petunia* wild-type (WT), scr_si*PhCRT3a*, and si*PhCRT3a* elongated pollen tubes. Both WT (**a**–**a’**) and scr_si*PhCRT3a* (**b**–**b’**) pollen tubes have a typical cylindrical shape with a clear zone in the growing tip; callose is uniformly distributed along the entire tube shank except at the elongating tip (**a”**,**b”**), respectively. In contrast, si*PhCRT3a* pollen tubes exhibit disturbed morphology (**c**–**h**) compared to control tubes, including misshapen tips (**c**,**d**,**d’**), twists in the shank (**c**–**h**), highly vacuolated cytoplasm (**d**–**f**) — even in the subapical domain (**d’**,**f’**) — and bifurcated tips (**e**–**g**) with one of them being dominant during elongation (**f**,**g**). Most of the si*PhCRT3a* pollen tubes exhibit increased callose deposition in the apical domain (**d”**,**f”**,**h”**) and in the twisted shank (**c”**). Finally, the growth of si*PhCRT3a* pollen tubes was inhibited, and some of them ruptured (**h**,**h’**). Bar: 50 µm (**a**–**h**); 20 µm (**a’**,**a”**–**h’**,**h”**).

First, we performed detailed light microscopic analysis of pollen tubes growing in the standard medium and in media supplemented with si*PhCRT3a* or scr_si*PhCRT3a* (Figure 5). In addition, to examine the effect of *PhCRT3a* knockdown on callose deposition, we used aniline blue to stain pollen tubes growing in different culture conditions. About 2 h after pollen germination in different culture media, cultivated pollen tubes appeared healthy and achieved comparable length. After this period, we observed the first signs of abnormal growth of si*PhCRT3a* pollen tubes, which intensified during the tubes’ elongation.

Elongated wild-type pollen tubes (WT, untreated tubes cultivated for about 4 h in standard medium) had a typical cylindrical shape with a clear zone in the growing tip (Figure 5a,a’). The control experiment showed that supplementation of the culture medium with scr_si*PhCRT3a* had no effect on the morphology or elongation of the pollen tubes (Figure 5b,b’). In contrast, si*PhCRT3a*-treated pollen tubes exhibited disturbed morphology during their elongation, and reduced length (Figure 5c–h) compared to WT and scr_si*PhCRT3a* tubes. Most of them showed similar morphological defects, including misshapen tips, twists in the shank, and highly vacuolated cytoplasm—even in the subapical zone and distal shank. All of these structural abnormalities were intensified during elongation, and correlated with a progressive loss of the clear zone at the growing pollen tube tip (Figure 5d’,f’,g’). Some of the growing si*PhCRT3a* pollen tubes exhibited bifurcated tube tips (Figure 5e), with one of them being dominant during elongation (Figure 5f,g). As expected, in most of the WT and scr_si*PhCRT3a* pollen tubes, callose was uniformly distributed along the entire tube shank, except at the elongating tip (Figure 5a’,a” and Figure 5b’,b”, respectively). We observed a similar pattern of callose distribution in the extended tip of the bifurcated si*PhCRT3a* pollen tubes (Figure 5e’,e”,g’,g”). However, in most of the si*PhCRT3a* pollen tubes we found increased callose deposition in their apical domain (Figure 5d’,d”,f’,f”), as well as in outgrowths of their twisted shanks (Figure 5c’,c”). Finally, the growth of si*PhCRT3a* pollen tubes was inhibited, and some of them ruptured after about 4–5 h of cultivation, releasing the cytoplasm (Figure 5h). These pollen tubes also exhibited abnormal callose deposition at the apex (Figure 5h’,h”).

**Figure 6:**
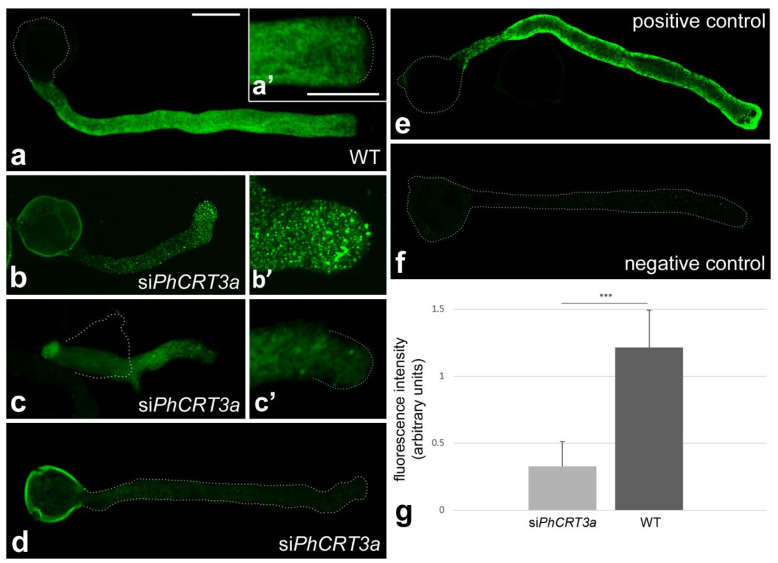
Quantitative analyses of *PhCRT3a* mRNA in WT (**a**) and si*PhCRT3a* (**b**–**d**) pollen tubes using fluorescent in situ hybridization (FISH). In WT tubes, the transcripts are diffusely distributed throughout the tube shank, extending from the base to the subapical zone (**a**,**a’**). In contrast, shorter si*PhCRT3a* pollen tubes exhibit a disrupted pattern of transcript localization (**b**,**b’**,**c**,**c’**), while elongated tubes are devoid of hybridization signals (**d**). Positive control—*PhCRT1* mRNAs in an elongated si*PhCRT3a* pollen tube (**e**). Negative FISH control without the molecular probe (**f**). The level of *PhCRT3a* transcripts is about 73% lower in si*PhCRT3a* pollen tubes compared to WT (**g**). Graphs show the relative *PhCRT3a* mRNA levels in pollen tubes elongating in different culture conditions (mean of 15 replicates for each experimental variant and standard deviation). Arbitrary units on the y-axis show total FISH intensity (pixels). Statistical analysis was carried out using the Mann–Whitney test (*** *p* ≤ 0.001). Bar: 50 µm (**a**–**f**); 20 µm (**a’**–**c’**).

**Figure 7:**
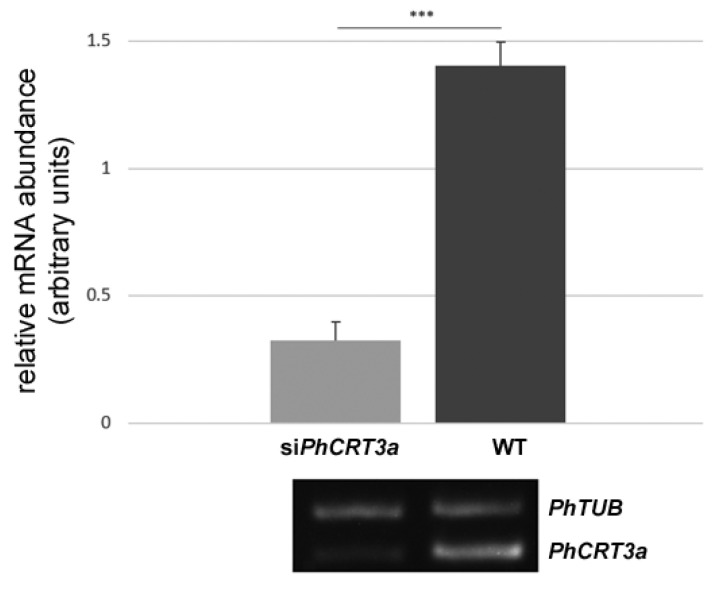
sqRT-PCR analysis of *PhCRT3a* mRNA levels in WT and si*PhCRT3a* elongated pollen tubes. The level of *PhCRT3a* transcripts is 77% lower in si*PhCRT3a* pollen tubes compared to WT tubes. Graphs show the relative *PhCRT3a* mRNA levels normalized to the *Petunia hybrida* tubulin (*PhTUB*) levels in pollen tubes elongating in different culture conditions (mean of 7 replicates for each experimental variant and standard deviation). Cropped representative agarose gel with *PhCRT3a* and *PhTUB* amplicons is shown under the graphs. Arbitrary units on the y-axis show total fluorescence intensity (pixels). Statistical analysis was carried out using the Mann–Whitney test (*** *p* ≤ 0.001). Representative gels are shown in Figure S2 in the Supplementary Materials.

To confirm that the morphological defects in growing pollen tubes were the result of post-transcriptional gene silencing (PTGS), we first performed fluorescent in situ hybridization (FISH) to detect *PhCRT3a* mRNA in pollen tubes growing in the standard medium and the medium supplemented with si*PhCRT3a* (Figure 6). In elongated WT pollen tubes (growing for about 4 h), these transcripts were diffusely distributed throughout the tube shank, extending from the pollen grain to the subapical zone of the tube (Figure 6a,a’). Shorter si*PhCRT3a* pollen tubes (growing for about 2.5–3 h after the addition of siRNA) exhibited a disrupted pattern of transcript localization from the base of the tube to the apical cytoplasm (Figure 6b,b’,c,c’), with numerous clusters of the FISH signal (Figure 6b,b’). In contrast, elongated si*PhCRT3a* pollen tubes (growing for about 4 h after the addition of siRNA) were nearly devoid of hybridization signals (Figure 6d). To test whether the used siRNA was sequence-specific, hybridization to *PhCRT1* mRNA in si*PhCRT3a* pollen tubes was performed. This positive control showed strong FISH signals in elongated si*PhCRT3a* pollen tubes (Figure 6e), and confirmed that silencing of the *PhCRT3a* gene was selective. As shown in Figure 6g, the level of *PhCRT3a* transcripts was about 73% lower in elongated si*PhCRT3a* pollen tubes than in elongated WT tubes. In addition, we performed sqRT-PCR to assess *PhCRT3a* mRNA levels in elongated WT and si*PhCRT3a*-treated *Petunia* pollen tubes. Our quantitative analysis confirmed that the level of *PhCRT3a* transcripts was about 77% lower in si*PhCRT3a* pollen tubes compared to WT tubes (Figure 7). Together, these results indicate that the morphological abnormalities observed during si*PhCRT3a* pollen tubes’ elongation are the result of PTGS, and that expression of the *PhCRT3a* gene is required for normal pollen tube growth in vitro.

### 2.3. Loss of PhCRT3a Causes Several Ultrastructural Defects in Elongating Pollen Tubes

**Figure 8:**
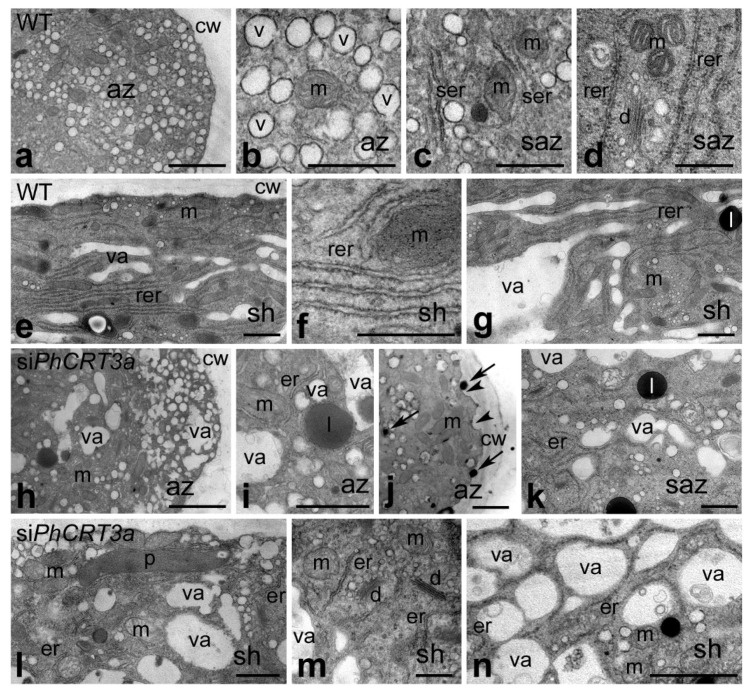
Ultrastructure of WT (**a**–**g**) and si*PhCRT3a*-treated (**h**–**n**) pollen tubes: In WT tubes, the apical zone (*az*; (**a**,**b**)) is packed with vesicles (*v*), and contains a few small organelles, such as mitochondria (*m*); the subapical zone (*saz*; (**c**,**d**)) contains many metabolically active organelles, such as mitochondria, dictyosomes (*d*), and well-developed ER structures (*er*), including rough ER (*rer*) and smooth ER (*ser*). Well-developed ER structures (mainly *rer*) are also present in the distal shank (*sh*; (**e**,**f**)), while the proximal shank is highly vacuolated. In si*PhCRT3a* pollen tubes, the apical zone (**h**–**j**) contains numerous mitochondria, very short or even fragmentized/disorganized ER, electron-dense vesicles (arrows in (**j**)), vacuoles (*va*), plastids (*p*), and lipid bodies (*l*); abnormal callose deposition is observed in the cell wall (*cw*) at the apex (arrowheads in (**j**)); in the subapical zone (**k**), only single small organelles, but numerous small vacuoles, are present; the distal shank (**l**–**m**) is highly vacuolated, and occasionally contains ER; however, numerous vesicles and small organelles such as mitochondria and dictyosomes are localized in this region of the tube; the proximal shank is highly vacuolated (**n**). Bar: 2 µm (**a**,**h**,**n**); 1 µm (**b**–**e**,**g**,**i**–**l**); 500 nm (**f**,**m**).

Given our observation that si*PhCRT3a*-treated pollen tubes exhibited several morphological abnormalities during elongation, we reasoned that si*PhCRT3a* tubes likely had ultrastructural defects and probably lost the cytoplasmic zonation typical of angiosperm pollen tubes. To explore this possibility, we performed high-resolution electron microscopy on ultrathin longitudinal sections of WT and si*PhCRT3a* elongated pollen tubes. WT pollen tubes showed the expected ultrastructural features (Figure 8a–g). The apical domain was clear and packed with vesicles (Figure 8a,b), and contained few small organelles such as mitochondria (Figure 8b). In the subapical zone, we observed numerous metabolically active organelles, including mitochondria (Figure 8c,d), well-developed rough/smooth ER (Figure 8c,d), and dictyosomes (Figure 8d). The shank contained extended ER cisternae (Figure 8e–g), mainly rough ER (Figure 8f), numerous small vacuoles (Figure 8e,g), and a few small organelles, such as mitochondria (Figure 8e–g). Finally, the proximal shank of the elongated WT pollen tubes was full of large vacuoles (Figure 8g).

Numerous aspects of pollen tubes’ apical domain ultrastructure were affected by knockdown of *PhCRT3a* expression (Figure 8h–n). This zone contained numerous vesicles of various sizes and irregular shapes, and even vacuoles (Figure 8h,i). We did not observe any obvious ultrastructural changes in the mitochondria in the apical zone, except that they tended to aggregate (Figure 8j). The ER cisternae at the tip were very short, indicating that they were highly fragmented/disorganized (Figure 8i). Additionally, numerous electron-dense vesicles (Figure 8j, arrows) and lipid bodies (Figure 8i) were visible in the apical domain of si*PhCRT3a*-treated pollen tubes. As si*PhCRT3a* pollen tubes grew, the number of electron-transparent vesicles decreased in the clear zone (Figure 8j), suggesting that vesicle trafficking was disrupted. Some of the si*PhCRT3a* pollen tubes had thicker cell walls at the apex compared to WT tubes (Figure 8j), and abnormal callose deposition was frequently observed in these tubes (Figure 8j, arrowheads). We also detected several defects in the subapical zone and the shank of abnormally growing si*PhCRT3a* pollen tubes (Figure 8k–n). In elongated si*PhCRT3a*-treated pollen tubes, ER cisternae were substantially shorter than in WT tubes (Figure 8k–m). Mitochondria and dictyosomes were not accumulated in the subapical cytoplasm (Figure 8k), but they were abundant in the shanks of these tubes (Figure 8l,m). We observed many small vacuoles and vesicles accumulated in the subapex, as well as lipid bodies (Figure 8k). Numerous electron-transparent vesicles were present in the distal shank of elongated si*PhCRT3a* pollen tubes (Figure 8l,m). Finally, the proximal shank of these tubes was highly vacuolated, and contained a few reduced ER structures and several mitochondria (Figure 8n). Together, our data confirm that *PhCRT3a* gene expression is required for normal vesicle trafficking, organelle transport/positioning, and ER ultrastructure in growing pollen tubes.

### 2.4. PhCRT3a Is Required for Normal F-actin Organization in Growing Pollen Tubes

**Figure 9:**
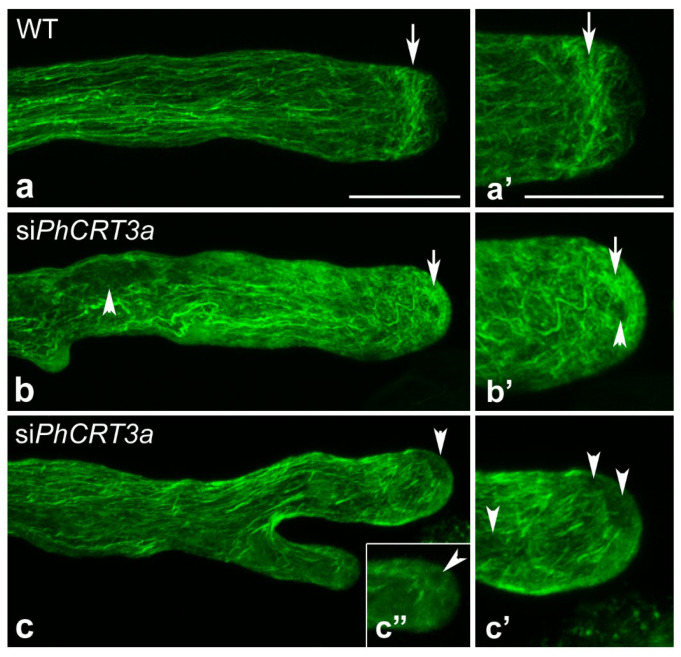
F-actin staining in WT and si*PhCRT3a* elongated pollen tubes. In WT pollen tubes, long actin filaments are distributed throughout the entire tube in a net axial array that is largely parallel to the direction of elongation, except at the very tip (**a**,**a’**), and a distinct cortical actin fringe is formed in the subapical zone of the tube (arrows in (**a**,**a’**)). By contrast, typical F-actin configurations are lost in the defined zones of si*PhCRT3a*-treated pollen tubes (**b**–**c”**), but abnormal actin aggregates are observed in the apical cytoplasm (arrows in (**b**,**b’**)). Additionally, some ‘‘empty spaces’’ (probably vacuoles) are present in the apical/subapical zone (arrowheads in (**b’**,**c**,**c’**,**c”**)), as well as in the distal shank (arrowhead in (**b**)), of si*PhCRT3a* pollen tubes. Bar: 50 µm (**a**–**c**); 20 µm (**a’**–**c”**).

Because knockdown of the *PhCRT3a* gene affected organelle positioning and vesicle transport, which are actin-dependent processes [32], we wanted to examine the effect of the loss of *PhCRT3a* on the arrangement of actin filaments in distinct cytoplasmic zones during pollen tube growth. First, we performed a comparative analysis of F-actin staining in pollen tubes elongated in different culture conditions (Figure 9). Our fluorescence staining confirmed that in the shank of WT pollen tubes actin filaments were typically organized in long actin bundles parallel to the long axis (Figure 9a). As expected from previous reports, these actin cables did not extend into the growing tip of the tube. Instead, we observed a distinct cortical fringe of densely packed actin filaments in the subapical zone (Figure 9a,a’, arrows), and only fine actin filaments in the tubes’ apical cytoplasm (Figure 9a’).

By contrast, in si*PhCRT3a*-treated pollen tubes, the actin filaments lost their specific configurations in defined cytoplasmic zones of the tube (Figure 9b,c). In the shank, long actin filaments were often twisted and thicker than in WT tubes (Figure 9b). In the presence of si*PhCRT3a* in the culture medium, the prominent actin fringe (normally existing behind the tube tip) was absent (Figure 9b’,c’). Instead of a fine meshwork of F-actin in the apical domain, we observed dense actin aggregates in si*PhCRT3a* pollen tubes (Figure 9b,b’, arrows). Moreover, we often observed ‘‘empty spaces’’ (probably vacuoles) in both the apical and subapical cytoplasm (Figure 9c,b’,c’,c”, arrowheads), as well as in the distal shank (Figure 9b, arrowhead) of elongated si*PhCRT3a* tubes. Abnormal actin configurations were also observed in bifurcated si*PhCRT3a* pollen tubes that lost the actin fringe in the subapical domain and the clear zone in the apex (Figure c–c”). Together, these results indicate that the loss of *PhCRT3a* affects the actin cytoskeleton’s organization in growing pollen tubes.

## 3. Discussion

Our previous work demonstrating that siRNA-mediated post-transcriptional silencing of the *PhCRT* gene belonging to the subgroup *CRT1/2* strongly impairs pollen tube elongation in vitro suggested that plant CRT plays a significant role in the tube growth [22]. Here, using the same experimental strategy, we confirm that diverse CRT isoforms are involved in the complex process of the pollen tubes’ apical growth.

### 3.1. PhCRT3a Is a Highly Conserved Gene Belonging to the Plant-Specific CRT3 Subfamily

Here, we report the first cloning of a new *CRT* gene — *PhCRT3a* — from the flowering plant *Petunia*. Alignment of the deduced amino acid sequence indicates that *Ph*CRT3a shares homology with other plant CRTs, and appears to be most similar to the plant-specific CRT3 from *Nicotiana* (87% identity). Indeed, our phylogenetic analysis indicates that the *PhCRT3a* cDNA clone that we identified belongs to the CRT3 subclass. This is consistent with the presence of an *N*-glycosylation site at position 107–110 aa. The location of this site near position 50–60 aa in the N-domain is shared among some plant CRT1/2 isoforms, whereas in CRT3 the *N*-glycosylation site is usually closer to 96 aa [16,19]. In *Arabidopsis* CRT3, the *N*-glycosylation site was estimated to be near position 102 aa [17,33]. Moreover, our cladogram analysis shows a clear distance between diverse subclasses of the CRT1/2 and CRT3 isoforms, confirming that *Ph*CRT3a is a member of the plant-specific subclass. Although multiple plant CRTs exist, several regions in their sequences are conserved, allowing them to maintain the basic functions of CRT related to Ca^2+^ homeostasis and quality control of newly synthesized glycoproteins within the secretory pathway. In fact, our in silico analysis of the deduced *Ph*CRT3a amino acid sequence revealed that the sequence contains several features typical of CRT family members: [16,17,18], including an ER-targeting signal in the N-terminus, the CRT family signature motifs 1 and 2 with cysteine residues that are important for proper folding of the protein, the three tandem repeats in the P-domain, and the ER retrieval signal in the C-terminus. However, comparing amino acid composition within the P- and C-domains between the *Ph*CRT1/2 [20] and *Ph*CRT3a isoforms, we found differences in the number of negatively charged residues for both of them. We determined that in *Ph*CRT3a the content of acidic residues was lower than in *Ph*CRT1/2, amounting to 21.6% (P-domain) and 29.7% (C-domain), whereas *Ph*CRT belonging to the subclass CRT1/2 contains 27.5% and 37% of acidic residues in the P- and C-domains, respectively [20]. It is believed that the main Ca^2+^-binding capacity of CRT proteins stems from the number of negatively charged amino acids in the C-terminus. Generally, CRT3 isoforms investigated in different plant species contain fewer acidic residues in the C-domain than CRT1/2 isoforms [16,17]. For example, *Arabidopsis* CRT1, CRT2, and CRT3 contain 37%, 35%, and 26% of the acidic residues in the C-domain, respectively [17]. Given the sequence features and our phylogenetic analysis, we can conclude that the *PhCRT3a* gene encodes a *Ph*CRT3 homolog.

### 3.2. Exogenous Delivery of PhCRT3a-Specific siRNA Effectively Impairs Pollen Tube Elongation

PTGS using specific siRNA is an essential method for studying the genomic functions of individual genes via loss-of-function experiments in plants. Proteome and transcriptome analyses have confirmed that the PTGS pathway components exist in many plant cell types, including pollen tubes, and experiments with transgenic plants expressing siRNA constructs indicate that these components are functional in pollen tubes [34,35]. Moreover, it was shown that the tip-growing germ tubes of fungal spores could take up siRNA directly from the culture medium [36], and the microspores of water ferns (*Marsilea*) were able to take up dsRNA from the medium at the time of their hydration [37]. Our previous [22] and present work demonstrated that *PhCRT*-specific siRNA was efficiently taken into cultured *Petunia* pollen tubes, causing a significant decrease in *PhCRT* mRNA levels without affecting the other genes. These results indicate that siRNA can overcome the barrier of the primary cell wall at the tube apex to enter the cytoplasm and induce the gene-silencing effect.

Here, our quantitative analyses clearly showed that *Petunia* pollen tubes growing in vitro took up si*PhCRT3a*, resulting in degradation of the *PhCRT3a* transcripts, as evidenced by greatly reduced FISH and sqRT-PCR signals in elongated si*PhCRT3a* pollen tubes. This efficient PTGS caused severe morphological and ultrastructural abnormalities in si*PhCRT3a*-treated pollen tubes, including loss of cytoplasmic zonation, disorganization of the actin cytoskeleton, and fragmentation/disorganization of the ER cisternae. In contrast, si*PhCRT3a* treatment did not affect the level of tubulin mRNA, confirming that si*PhCRT3a* selectively knocked down *PhCRT3a* gene expression. Although the si*PhCRT3a* pollen tubes eventually ruptured, we do not interpret this as a result of stress, for two reasons: First, the tubes continued growing for up to 4–5 h. Second, although we observed incorrect callose deposition in elongated si*PhCRT3a* pollen tubes, we did not find enhanced callose deposition at the whole tube tips, which is as well-characterized response to toxic conditions [13]. Callose is one of the major cell wall components in the pollen tube shank (Figure 1). This plant polysaccharide is synthesized by callose synthase, whose position in the plasma membrane is coordinated by actin filaments [38]. We demonstrated that the loss of *PhCRT3a* strongly impairs F-actin organization in *Petunia* pollen tubes, particularly in the subapical and apical domains. Thus, disorganization of the actin cytoskeleton in these zones can result in disturbed localization of the callose synthase in the tube plasma membrane and, consequently, enhanced callose deposition in response to si*PhCRT3a* treatment. On the other hand, callose deposition at the pollen tube tip is induced by a sudden increase in cytoplasmic Ca^2+^ levels in the apical cytoplasm [39]. We previously showed that in *Petunia* pollen tubes growing in vitro, CRT is translated on ER-membrane-bound ribosomes that are abundant in the subapical zone, where CRT’s Ca^2+^-buffering and chaperone activities might be particularly required during extremely fast pollen tube elongation [15]. Given that all of the CRT family members play an important role in regulating Ca^2+^ homeostasis, the lack of one of them may result in elevated cytosolic Ca^2+^ in the tube cytoplasm. Although CRT3 isoforms have a reduced Ca^2+^-binding capacity compared to CRT1/2 proteins [16,17], CRT3 isoforms retain their basic CRT properties and functions related to Ca^2+^ homeostasis and quality control of newly synthesized glycoproteins in the ER.

### 3.3. CRT Is Required for Polarized Pollen Tube Cytoplasm and Functional Organization of the Actin Cytoskeleton

Pollen tubes cultured in standard media usually exhibit a cylindrical, uniaxial structure with highly polarized cytoplasmic organization, including a vesicle-packed clear zone and an organelle-rich subapical domain (Figure 1). Here, we show that knockdown of *PhCRT3a* strongly affected *Petunia* pollen tubes’ morphology and ultrastructure. We observed twisted si*PhCRT3a* pollen tubes with misshapen or bifurcated tips. One of the most obvious ultrastructural phenotypes was that numerous mitochondria, ER, lipid bodies, clusters of vesicles, and even vacuoles penetrated the tube’s apex, leading to a loss of the clear zone. These structural abnormalities were intensified during the pollen tube elongation. There is a consensus that the actin cytoskeleton supports the development and maintenance of pollen tube shape and its polarized cytoplasm, because actin filaments have a major influence on various processes, such as vesicle/organelle transport, as well as endo- and exocytosis [7,8,9,32]. In addition, the actin fringe in the subapical zone of the tube is believed to function as a ”stop-and-go” point for organelles transported to the apex that filters organelles and allows only secretory vesicles containing new cell membrane and cell wall resources to move to the clear zone. This allows pollen tubes to elongate into a cylindrical shape. In contrast, when the functional organization of the actin cytoskeleton is affected, transport vesicles are not focused properly, and they may fuse into clusters, causing excessive apex expansion and irregular growth [7]. Within the pollen tubes, the assembly and disassembly of the actin filaments are promoted by many actin-binding proteins, including nucleating, depolymerizing, severing, capping, F-actin-stabilizing, and G-actin-sequestering proteins [9,40]. In addition, studies on pollen tubes from different plant species have also confirmed that organelle movement and apical accumulation of vesicles depend on the actomyosin system, including class XI myosins [41].

Our present work provides evidence that *PhCRT3a* knockdown causes disorganization of F-actin structures in distinct cytoplasmic zones of *Petunia* pollen tubes—particularly in the subapical and apical zones. This observation is not surprising, as the thicker F-actin bundles in the tube shank are relatively stable, but the thinner actin filaments in the growing domain are highly dynamic [42]. We previously demonstrated that the loss of function of *PhCRT* belonging to the *CRT1/2* subclass strongly impairs F-actin organization and function in *Petunia* pollen tubes in both the growing domain and the shank [22]. Therefore, we argue that diverse CRT isoforms are involved (and possibly co-operate) in the functional organization of the actin cytoskeleton, which is strictly required for the key processes driving pollen tube tip growth. We propose that the mechanism underlying CRT’s function in pollen tube elongation involves the activity of various actin-binding proteins [7,9,13,40]. These actin-binding proteins have a uniform distribution throughout the tube. However, each of them is sensitive to different Ca^2+^ concentrations. For example, plant villin can bundle actin filaments at lower Ca^2+^ concentrations in the tube shank, while it serves and caps microfilaments under a higher concentration of Ca^2+^ in the pollen tube tip. Profilin binds G-actin and regulates F-actin polymerization. However, in pollen tubes’ apical region with high Ca^2+^ concentration, this protein sequesters G-actin and prevents its polymerization into F-actin. Additionally, elevated Ca^2+^ levels can inhibit myosin XI ATPase activity and, thus, impair the motility of organelles and vesicles [43]. The subapex of *Petunia* pollen tubes, where the Ca^2+^ concentration is much lower than in the apical cytoplasm, is rich in rough/smooth ER ([15,22], and this work). Therefore, we propose that diverse CRT isoforms are translated on ER-membrane-bound ribosomes, enabling them to sequester exchangeable Ca^2+^ in the subapical zone of the tube. In the absence of these Ca^2+^-buffering proteins, the Ca^2+^ homeostasis in growing pollen tubes may be destabilized; as a result, we observed disorganization of the unique F-actin structures in both si*PhCRT1/2* [22] and si*PhCRT3a* pollen tubes. It should be noted that si*PhCRT1/2* treatment strongly impairs F-actin organization in both the growing domain and the shank of *Petunia* pollen tubes [22], while si*PhCRT3a* pollen tubes primarily showed disorganization of the microfilaments in the apical/subapical domains. Moreover, si*PhCRT1/2* treatment caused destabilization of the Ca^2+^ gradient in *Petunia* pollen tubes [22], and we did not confirm this in si*PhCRT3a* tubes. These data prove that the silencing of *PhCRT1/2* causes severe destabilization of Ca^2+^ homeostasis in the pollen tube cytoplasm, while the loss of *PhCRT3a* slightly affects local Ca^2+^ levels in the growing domain of the tube. This supports *Ph*CRT1/2 as being the main isoform, possibly due to an enhanced Ca^2+^-binding efficiency, and may indicate a less dominant role for *Ph*CRT3a in Ca^2+^ homeostasis. Actin polymerization is necessary for secretory vesicles to accumulate in the apical inverted cone, but actin depolymerization is required for vesicle docking and fusion at the plasma membrane [40]. Additionally, vesicle trafficking/docking in the growing tip of the pollen tube correlates directly with oscillatory changes in apical Ca^2+^ concentration. Here, we show that knockdown of *PhCRT3a* in *Petunia* pollen tubes results in apical F-actin accumulation and progressive loss of the clear zone. Taken together, we can conclude that diverse CRT isoforms indirectly affect the functional organization of the actin cytoskeleton and, thus, pollen tube elongation.

### 3.4. CRT Is Crucial for ER Structure and Function in Growing Pollen Tubes

One of the typical ultrastructural features of growing pollen tubes is the enrichment of the rough and smooth ER in the subapical zone, whereas rough ER is abundant, starting behind the inverted cone of vesicles throughout the rest of the cytoplasm [2,3]. Here, we show that the rough ER co-localized with *PhCRT3a* mRNA in the subapex and the shank of *Petunia* pollen tubes, and the smooth ER enriched the subapical domain. Previously, the same results we confirmed for *PhCRT1/2* mRNA; however, we found that *18S* rRNA and CRT protein (unidentified isoform) were preferentially localized in the subapical domain of highly elongated *Petunia* pollen tubes [15,22]. Thus, we can conclude that diverse CRT isoforms are translated on ER-membrane-bound ribosomes and accumulated in the abundant ER at the subapical cytoplasm of growing pollen tubes. The intense CRT labeling (unidentified isoform) at the subapical zone has also been reported in *Nicotiana*, *Haemanthus*, and *Petunia* pollen tubes [44,45,46,47]. Knockdown of *PhCRT3a* (present work) as well as *PhCRT1/2* (previous work, [22]) in elongating *Petunia* pollen tubes strongly disturbed the ER localization and structure. The ER cisternae in both si*PhCRT3a* and si*PhCRT1/2* pollen tubes were highly fragmented and disorganized. Reduction in CRTs prevented the specific ER accumulation in the subapex of elongated pollen tubes, and may explain why many of the ER cisternae were instead observed in the clear zone. In si*PhCRT3a* as well as si*PhCRT1/2* pollen tubes, protein folding may be impaired, leading to the accumulation of unfolded proteins in the ER. These dysfunctional proteins may, in turn, disturb the ER ultrastructure. Studies by Jin et al. [48] revealed that a plant-specific CRT3 isoform in *Arabidopsis* is a key retention factor for a defective brassinosteroid receptor in the ER. They argue that the CRT1/2 family members might be the most important for ER Ca^2+^ homeostasis as a result of their acidic C-terminal tails containing low-affinity/high-capacity Ca^2+^-binding sites, whereas the divergent CRT3 member is mainly responsible for retaining misfolded glycoproteins in the folding compartment—especially under stress conditions. When the pollen tube grows inside the pistil, carrying male gametes to the ovule, it invades diverse tissues, sensing and adapting to abrupt transitions in the mechanical environment. Thus, the role of the ER and the ER molecular chaperones in response to stress appears to be crucial in the development and elongation of the pollen tube. On the other hand, the extremely fast tip growth of the pollen tube requires very high rates of protein synthesis and the quality control of glycoproteins passing through the ER. The *Ph*CRT3a isoform may play a particularly important role in these processes, since it has a reduced Ca^2+^-binding capacity compared to CRT1/2 proteins. Future studies will address this possibility.

## 4. Materials and Methods

### 4.1. Plant Material and Pollen Tube Cultures

Freshly collected pollen of *Petunia hybrida* (commercial cultivars grown at room temperature) was germinated in liquid culture medium containing 0.2% sucrose, 0.05% Ca(NO)_3_, 0.01% MgSO_4_, 0.01% H_3_BO_4_, 0.01% KNO_3_, 15% polyethylene glycol 4000, and 0.4% 2-(N-morpholino) ethanesulfonic acid (pH 6.0). For PTGS experiments, the culture medium was supplemented with (1) *PhCRT3a*-specific siRNA (si*PhCRT3a*) or (2) scrambled siRNA (scr_si*PhCRT3a*, negative control), and the cultures were incubated at 30 °C for about 4 h. Pollen tubes growing in either the standard medium or medium supplemented with si/scrRNAs were prepared for detailed morphological observations using light microscopy, sqRT-PCR analysis, FISH, F-actin staining, and electron microscopy, as described below. For each experiment, pollen collected from mature anthers at dehiscence was mixed in the following ratio: pollen grains from five anthers/2 mL liquid culture medium. For RACE and RT-PCR, pollinated *Petunia* pistils dissected from open flowers were used. All experiments were repeated at least three times, with similar results.

### 4.2. RNA Extraction

For RT-PCR, plant material (100 mg of pistils) was ground in liquid nitrogen, while for sqRT-PCR, elongated in vitro pollen tubes were collected from the culture medium. Total RNAs were isolated with RNA Extracol^®^ (EURx) and treated with DNase I, RNase-free (Thermo Fisher Scientific, Waltham, MA, USA) according to the manufacturer’s protocols. Samples were subsequently extracted with one volume of acid–phenol:chloroform:isoamyl alcohol mixture (125:24:1, Merck, Darmstadt, Germany), and then with one volume of chloroform. Isopropanol-precipitated RNAs were pelleted at 15,000× *g* for 15 min (4 °C) and washed with ice-cold 70% ethanol. Concentrations of purified RNAs were measured with a NanoDrop One spectrophotometer (Thermo Fisher Scientific, Waltham, MA, USA), and the quality of each sample was additionally assessed by visualization on a 1% agarose gel in 1 × TBE buffer.

### 4.3. Random Amplification of cDNA Ends (RACE) and Amplification of the Full-Length PhCRT3a cDNA

For 3′ and 5′-RACE, gene-specific primers were based on a partial *CRT3* cDNA sequence of *Petunia inflata* (Peinf101Scf01985g01011.1) published in the Sol Genomics Network. 5′-RACE was carried out using the FirstChoice RLM-RACE kit (Thermo Fisher Scientific, Waltham, MA, USA), according to the manufacturer’s protocol. Total RNA isolated from *Petunia* pistils was dephosphorylated with calf intestinal phosphatase and treated with tobacco acid pyrophosphatase to remove the 5′ cap structure from the full-length mRNA. The 5′-RACE Adapter was ligated to treated mRNA with T4 RNA ligase. Reverse transcription was performed with random decamers in the presence of the M-MLV enzyme and RNase inhibitor at 42 °C for 1 h. A 1 μL aliquot of the reverse transcription reaction was used in the outer 5′-RACE PCR in the presence of the 5′-RACE outer primer, 5′-RACE gene-specific outer primer, and Platinum™ SuperFi™ DNA Polymerase (Table 1). PCR cycles were as follows: 95 °C for 30 s, followed by 35 cycles of 95 °C for 30 s, 59 °C for 30 s, and 72 °C for 30 s, followed by a final extension step of 72 °C for 4 min. A 1 μL aliquot of the outer 5′-RACE PCR mixture served as the template in an inner 5′-RACE PCR using the 5′-RACE inner primer and 5′-RACE gene-specific inner primer (Table 1). PCR cycles were as above.

3′RACE was carried out using the 5′/3′ RACE Kit (Merck, Darmstadt, Germany) according to the manufacturer’s protocol. Reverse transcription was performed with the Transcriptor Reverse Transcriptase and Oligo dT-Anchor Primer in the presence of RiboLock RNase Inhibitor (Thermo Fisher Scientific, Waltham, MA, USA) at 55 °C for 1 h. A 1 μL aliquot of the reverse transcription reaction was then used in the outer 3′-RACE PCR with the PCR anchor primer and 3′-RACE gene-specific outer primer (Table 1), along with Platinum™ SuperFi™ DNA Polymerase. PCR cycles were as follows: 95 °C for 30 s, followed by 35 cycles of 95 °C for 30 s, 59 °C for 30 s, and 72 °C for 30 s, followed by a final extension step of 72 °C for 4 min. A 1 μL aliquot of the outer 3′-RACE PCR mixture served as the template in the inner 3′-RACE PCR using the PCR anchor primer and 3′-RACE gene-specific inner primer (Table 1). PCR cycles were as above. The 3′- and 5′-RACE PCR products were visually inspected on a 1% agarose gel in 1 × TAE buffer.

RT-PCR was carried out to amplify the full-length *PhCRT3a* cDNA sequence. First-strand cDNA synthesis was performed with 2.5 μg of total RNA extracted from *Petunia* pistils using the NG dART RT kit and an oligo(dT)_20_ primer, according to the manufacturer’s instructions (EURx). A 1 μL aliquot of the reverse transcription reaction was used as the template for PCR amplification with Platinum^TM^ SuperFi™ DNA Polymerase (Thermo Fisher Scientific, Waltham, MA, USA) and the outer gene-specific primers (Table 1). A 1 μL aliquot of the first PCR mixture served as the template in a second PCR using the inner gene-specific primmer (Table 1). PCR cycles were as follows: 95 °C for 90 s, followed by 35 cycles of 95 °C for 30 s, 60 °C for 30 s, and 72 °C for 60 s, followed by a final extension step of 72 °C for 4 min. The RT-PCR products were visually inspected on a 1% agarose gel in 1 × TAE buffer.

### 4.4. siRNA and scrRNA Oligonucleotides Used in PTGS Experiments

The full-length *PhCRT3a* cDNA sequence was analyzed using the Eurofins Genomics siRNA design tool (Eurofins Genomics, Louisville, KY, USA). Predicted target regions found in a BLAST search (http://blast.ncbi.nlm.nih.gov/Blast.cgi, assessed on 19 November 2020) to contain more than 14 contiguous bases pairs of homology to other *Petunia* genes in the NCBI database were excluded from further analysis. Based on the above results, three different antisense siRNAs were selected for analysis in SSEARCH (http://www.ebi.ac.uk/services/dnarna, assessed on 19 November 2020) to determine their specificity to *PhCRT3a* mRNA. The seed regions of each siRNA were aligned with sequences in the miRBase sequence database (https://www.mirbase.org/index.shtml, assessed on 11 December 2020). Sequences without similarity to miRNAs of *Solanaceae* representatives placed in the database were used as a template for siRNA synthesis. Additionally, siRNAs were confirmed to not include tracts of more than four consecutive homonucleotides, and to have a G/C of no more than 38.0%. As an initial test of these three siRNAs, we supplemented pollen tube culture medium with various concentrations of siRNA duplexes, and growing pollen tubes were observed at different time points (every 30 min). Preliminary observations using stereo/light microscopy revealed the most effective siRNA (si*PhCRT3a*, Table 1) that affected pollen tube growth, giving the same phenotypes of elongating tubes at all of the concentrations tested. Therefore, we finally used this si*PhCRT3a* duplex at the lowest effective concentration (1.5 µM) in all subsequent PTGS experiments. In addition, a scrambled version of si*PhCRT3a* that did not target any gene was used as the negative control (scr_si*PhCRT3a*, Table 1). Each oligo was modified at the 3′ end with two [dT] nucleotides to increase nuclease stability [49], and HPLC was purified and delivered in duplex format (sense and antisense strands) by Merck. Both of the above si/scrRNAs were added separately to the culture media at the same final concentrations.

### 4.5. sqRT-PCR Analysis and Quantification

For sqRT-PCR, first-strand cDNA synthesis was performed with 2.5 μg of total RNA extracted from WT and si*PhCRT3a* pollen tubes using the NG dART RT kit and an oligo(dT)_20_ primer according to the manufacturer’s instructions (EURx). Then, 1 μL of first-strand cDNA was used as a template for PCR amplification with Platinum™ II Taq Hot-Start DNA Polymerase (Thermo Fisher Scientific, Waltham, MA, USA) and gene-specific primers (Table 1). Both pairs of gene-specific primers were based on the partial *CRT3* cDNA sequence of *Petunia axillaris* (Peaxi162Scf00858g00216.1) or the tubulin EST sequence (EST885118) from *Petunia hybrida* published in the Sol Genomics Network (SGN) or GDB, respectively. PCR cycles were as follows: 95 °C for 90 s, followed by 33 cycles of 95 °C for 30 s, 60 °C for 30 s, and 68 °C for 30 s, followed by a final extension step of 68 °C for 4 min. For each pair of primers, the PCR conditions—including the concentrations of primers, DNA polymerase, and Mg^2+^, the annealing temperature, and the number of cycles—were optimized as described in [50]. Fluorescence signals of both *PhCRT3a* and *PhTUB* amplicons resolved in 2% agarose gel in 1 × TAE buffer were acquired using the ChemiDoc™ Touch Imaging System (Bio-Rad, Hercules, CA, USA) and quantified with Image Lab 6.1 software (Bio-Rad, Hercules, CA, USA). All data obtained from the sqRT-PCR experiments were subjected to the Mann–Whitney test.

### 4.6. Plasmid Construction, Molecular Probe Synthesis, and FISH

Nested RT-PCR or RACE products were recombined into the pCR4 Blunt-TOPO vector using the DNA topoisomerase I enzyme, according to the protocol provided with the Zero Blunt™ TOPO™ PCR Cloning Kit for Sequencing Kit (Thermo Fisher Scientific, Waltham, MA, USA). The insert was verified by sequencing for its correct amplification and orientation in pCR4. To prepare the full-length *PhCRT3a* antisense probe, the plasmid containing the *PhCRT3a* insert was cut with Xba I and purified on CHROMA SPIN™ + TE − 100 columns. A 1 μg aliquot served as a template to generate the digoxigenin (DIG)-labeled probe using the DIG RNA Labeling Kit (SP6/T7), according to the manufacturer’s instructions (Merck, Darmstadt, Germany). The molecular probe was further used in FISH. The *PhCRT1* antisense probe was generated using a cloned cDNA sequence—HG738129 [20].

Elongated WT and si*PhCRT3a* pollen tubes were fixed in freshly prepared 4% formaldehyde (Polysciences, Warrington, PA, USA) in PBS (pH 7.2), and then enzymatically digested using a mixture of 1% (*w*/*v*) cellulase R10 (Serva) and 27 U of pectinase (Merck, Darmstadt, Germany) per mL in 0.01 M citrate buffer (pH 4.8) for 25 min at 37 °C. Next, the pollen tubes were permeabilized with 0.1% saponin in PBS (pH 7.2). mRNA *PhCRT3a* transcripts were localized with the DIG-labeled molecular probe at a final concentration of 1.25 µM. Pre-hybridization and hybridization were carried out in 50% formamide, 4 × SSC, 5 × Denhardt’s solution, 1 mM EDTA, and 50 mM sodium phosphate buffer (pH 7.0). Hybridization signals were detected using primary mouse anti-DIG (Roche, Basel, Switzerland) and secondary anti-mouse-Alexa Fluor 488 (Invitrogen, Waltham, MA, USA) antibodies, and a no-probe negative control was also performed. Specimens were covered with ProLong Gold mounting medium (Life Technologies, Carlsbad, CA, USA) to prolong the fluorescence signals. FISH images were acquired using the software package LAS AF (Leica, Wetzlar, Germany) connected to a Leica SP8 confocal microscope with a 63 × (numerical aperture, 1.4) Plan Apochromat DIC H immersion oil lens.

For quantitative measurements, each FISH experiment was performed with consistent experimental conditions and concentrations of the molecular probe, primary and secondary antibodies, and a small pinhole and 75 ls exposure time were used for all analyzed samples. Three-dimensional optical sections of pollen tubes were acquired with at 1.0 µm step intervals, from a minimum of 20 comparable pollen tubes. All data were corrected for background autofluorescence as determined by signal intensities in negative controls. For image processing and analysis, ImageJ (NIH, Bethesda, MD, USA) software was used. For signal evaluation, Cell Statistical Analyser and Image Gauge 3.46 Fujifilm software were used. The total fluorescence intensity was measured per single pollen tube. PAST 3 software and Microsoft Excel (Microsoft, Redmond, Washington, DC, USA) were used for statistical analysis, and the statistical significance of data was determined using the Mann–Whitney test.

### 4.7. F-actin Staining

In vitro elongated WT and si*PhCRT3a* pollen tubes were transferred to liquid culture medium containing 400 μM m-maleimidobenzoyl-N-hydroxysuccinimide ester (Merck, Darmstadt, Germany) for 6 min. Next, pollen tubes were permeabilized with an actin-stabilizing buffer (ASB) composed of 100 mM PIPES, 5 mM MgSO_4_, 0.5 mM CaCl_2_, and 0.05% Triton X-100 (pH 9.0) for 5 min, and fixed with freshly prepared 2% formaldehyde in ASB at room temperature for 30 min. Pollen tubes were washed three times with ASB as above, except at pH 7.0, and supplemented with 10 mM EGTA (Merck, Darmstadt, Germany). After washing, the samples were labeled with 1 μM AlexaFluor^TM^488 Phalloidin (Invitrogen, Waltham, MA, USA) ASB-EGTA without Triton X-100 for 30 min in the dark. Finally, labeled pollen tubes were transferred to microscope slides and covered with ProLong Gold mounting medium (Life Technologies, Carlsbad, CA, USA), and images of microfilaments were acquired using the software package LAS AF connected to a Leica SP8 confocal microscope with a 63× (numerical aperture, 1.4) Plan Apochromat DIC H immersion oil lens. For image processing and analysis, ImageJ (NIH, Bethesda, MD, USA) software was used.

### 4.8. Light and Transmission Electron Microscopy

Cultivated WT, si*PhCRT3a*-treated, and scr_si*PhCRT3a*-treated pollen tubes were fixed with 2% glutaraldehyde (Merck, Darmstadt, Germany) in PBS (pH 7.2) for 2 h at room temperature. Next, samples were washed three times in PBS, placed on microscope slides, and observed under the light microscope. To identify morphological defects caused by PTGS, pollen tubes were fixed every 30 min during the 4 h culture and then examined by light microscopy. For callose imaging, elongated pollen tubes were stained with 0.1% aniline blue according to the standard protocol and observed using a Nikon Eclipse 80i fluorescence microscope.

For detailed ultrastructural analysis, elongated WT and si*PhCRT3a* pollen tubes were fixed with 2% glutaraldehyde (Merck, Darmstadt, Germany) in PBS (pH 7.2) for 2 h at room temperature, followed by overnight at 4 °C, and then post-fixed with 2% osmium tetroxide (Merck, Darmstadt, Germany) in PBS for 30 min at room temperature. Samples were rinsed with PBS and Milli-Q-filtered water, dehydrated in ethanol, and embedded in Poly/Bed 812 resin (Polysciences, Warrington, PA, USA) according to the standard protocol. Ultrathin longitudinal sections of elongated pollen tubes were post-stained with uranyl acetate and lead citrate solutions before observation using a JEOL JEM 1010 transmission electron microscope.

### 4.9. In Silico Sequence Analysis, Sequence Selection, and Phylogenetic Analysis

The expected amino acid sequence of the *Ph*CRT3a was identified with the Expasy translate tool (https://web.expasy.org/translate/, assessed on 4 September 2019). Potential molecular weights and isoelectric points were predicted by applying the Compute pI/Mw tool (https://web.expasy.org/compute_pi/, assessed on 29 October 2019). Analysis of structural motifs was carried out using available software, including MotifScan (https://myhits.sib.swiss/cgi-bin/motif_scan, assessed on 4 September 2019), InterPro (https://www.ebi.ac.uk/interpro/, assessed on 4 September 2019) and NetNGlyc (https://services.healthtech.dtu.dk/service.php?NetNGlyc-1.0, assessed on 4 September 2019). In addition, the prediction of *Ph*CRT3a motifs was carried out based on a comparative assay with the literature data.

The plant cDNAs, representing different CRT subclasses, were obtained from the National Center for Biotechnology Information (NCBI; http://www.ncbi.nlm.nih.gov, assessed on 3 September 2019) and published papers [16,28,29,30]. Each cDNA was verified and identified based on the annotation from the database, and comprised both 5′ and 3′ untranslated regions (UTRs) and open reading frames (ORFs). Moreover, the received putative polypeptides consisted of sequence flanked by the Met and STOP codons. The identity level and alignment of the predicted amino acid sequences were determined using Clustal software (Clustal Omega, https://www.ebi.ac.uk/Tools/msa/clustalo/, assessed on 4 September 2019). Next, the alignment was used to construct the rooted phylogenetic tree with Mega X v.10.1.7 [51]. The neighbor-joining algorithm with the Poisson correction model was applied. The bootstrap value was received by 1000 replications with a 70% cutoff level on the branches [31]. The tree was rooted by the *Selaginella moellendorffii* CRT sequence (GenBank accession number XP_002968331.2). The NCBI accession number of each sequence is shown after an underscore. 

## 5. Conclusions

Taken together, our data indicate that *Ph*CRT3a is a one of the key regulators involved in multiple aspects of pollen tube growth, including regulation of the actin cytoskeleton’s arrangement/function, control of secretion, and maintenance of ultrastructure. We suggest that *Ph*CRT3a’s molecular chaperone and Ca^2+^-buffering activities facilitate the tube elongation — a process that requires both high rates of protein synthesis and stabilization of Ca^2+^ homeostasis. Currently, the role of a variety of ER chaperones — including CRT isoforms — in pollen tube development and functioning is under heated debate, and opposing arguments are put forward by different research groups. Therefore, further characterization of *Ph*CRT3a-dependent processes and the extent of crosstalk with other important pathways and molecules is required to fully understand the molecular mechanism/s by which this protein acts to control pollen tube growth.

## Figures and Tables

**Table 1:** List of sequences used in RACE, sqRT-PCR, RT-PCR, and PTGS experiments.

**RACE**	3′-RACE—GSP Outer primer	5′CTAATAGGGAGGCTGAAAAGGA3′
	3′-RACE—GSP Inner primer	5′GCAGAGAAAGTGAGAAAAGCAA3′
	5′-RACE—GSP Outer primer	5′GCCACCACACTCAATGTCTTGT3′
	5′-RACE—GSP Inner primer	5′AATGTTTGGCATCGGTTGAC3′
**sqRT-PCR**	*PhCRT3a*	5′GAGACAGAAAGCCAGCTTCATT3′ 5′ACGCCTCCTCTAGAAAGGTTTT3′
	*PhTUB*	5′CTAGAGGTCTCTCAATGGCATC3′ 5′TCCTCCTCATCCTCATATTCAC3′
**RT-PCR**	Full-length *PhCRT3a* gene-specific primers	5′AAAATTCACAGTAATGAGCAAATGGCTC3′ 5′CGACTGAAGAGTAATTTTTTTCGATTTATTTATC3′
**PTGS experiments**	si*PhCRT3a* scr_si*PhCRT3a*	5′GCAGGAUCAUUUAAGCACA[dT][dT]3′ 5′GCUACAGAUUGCGUAACAA[dT][dT]3′

## Data Availability

The data that support the present results are available from the corresponding author upon reasonable request.

## Supplementary Materials

The following supporting information can be downloaded at: https://www.mdpi.com/article/10.3390/ijms23094987/s1.
