# Supplementary figures and images for: RNAi-Mediated Knockdown of Calreticulin3a Impairs Pollen Tube Growth in Petunia
Source: Int J Mol Sci. 2022 Apr 30;23(9):4987. doi: 10.3390/ijms23094987 (PMC9103332; doi:10.3390/ijms23094987)

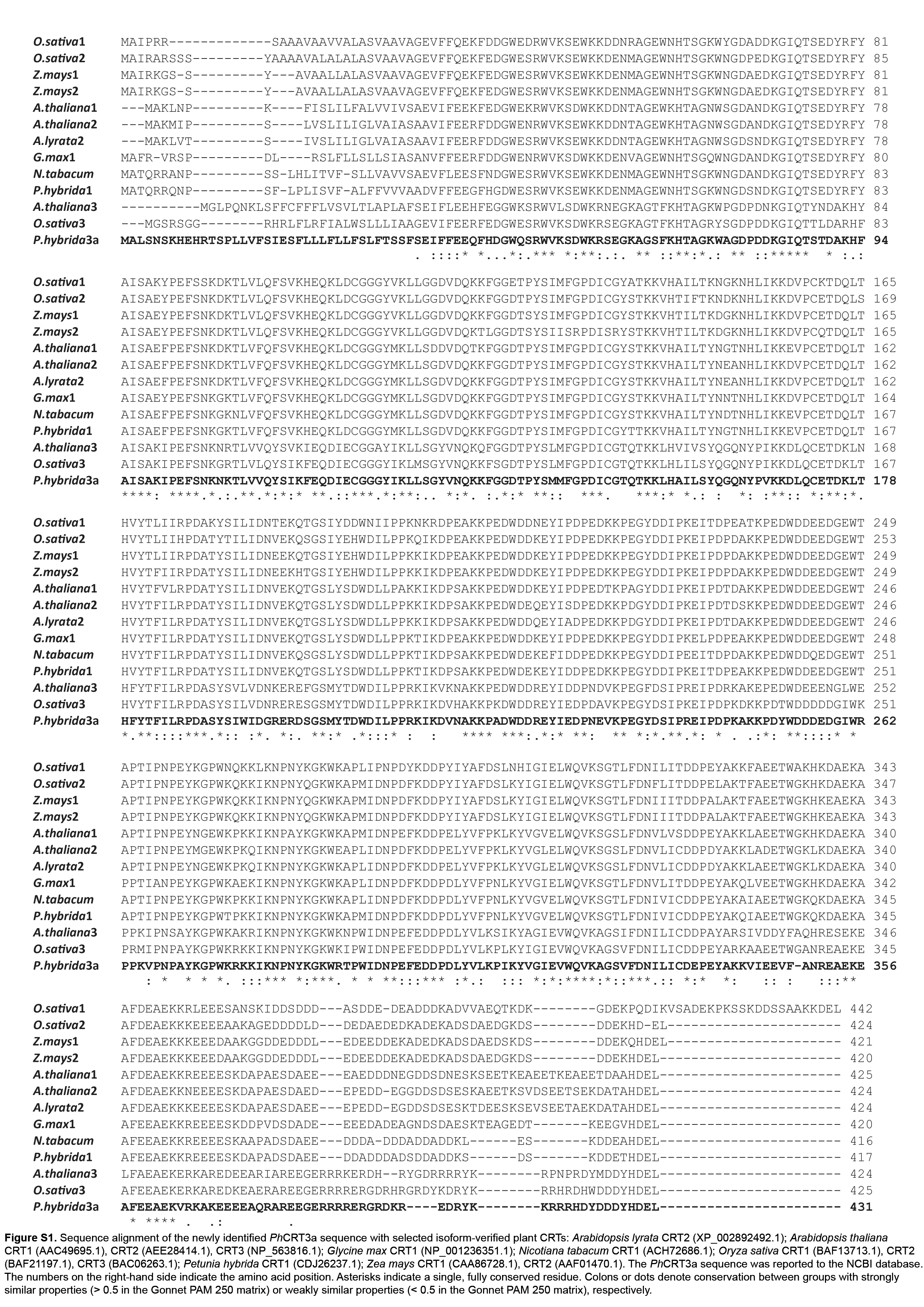

Supplement: Supplementary file 1 [file ijms-23-04987-s001.zip › Figure S1.tif]

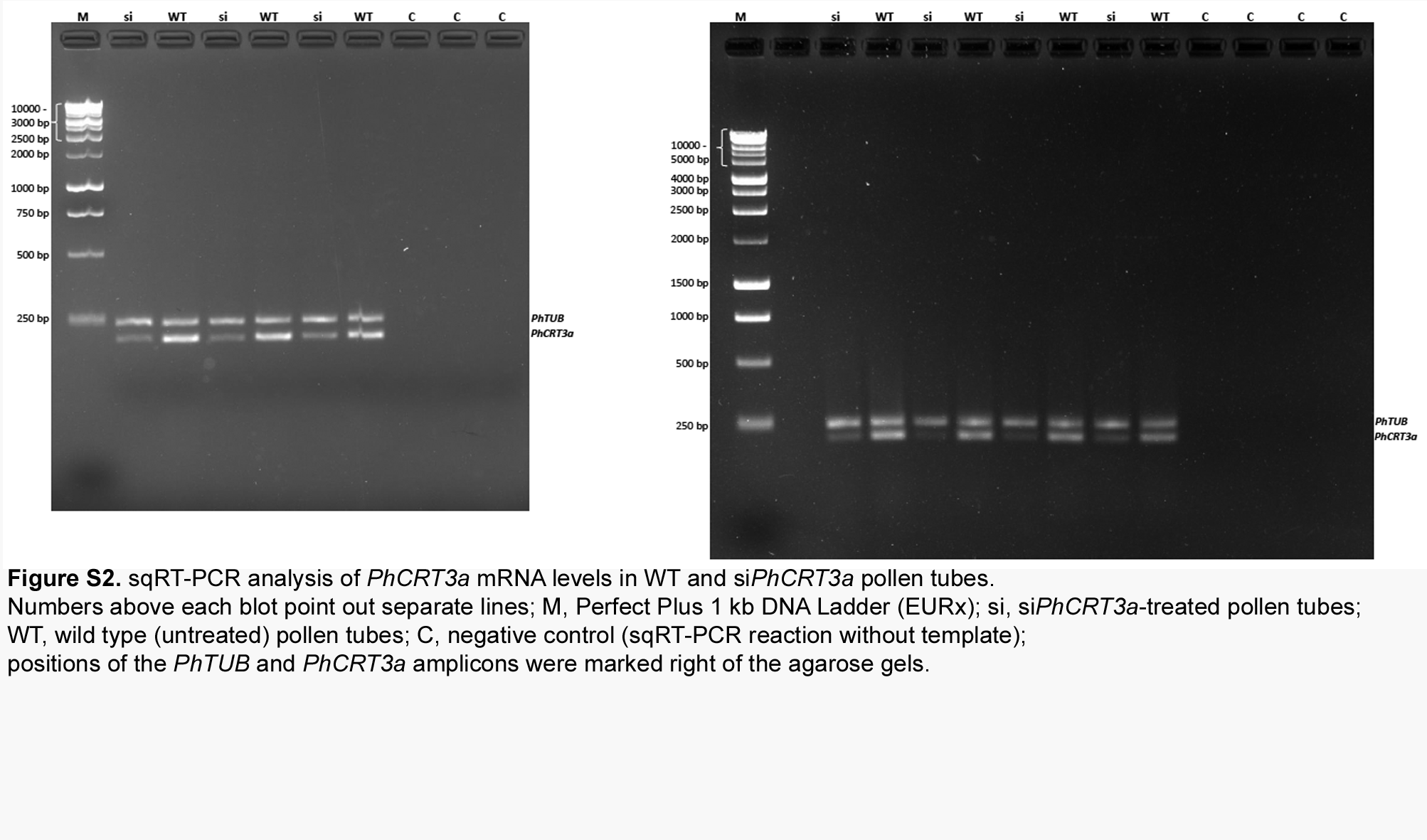

Supplement: Supplementary file 1 [file ijms-23-04987-s001.zip › Figure S2.tif]
